# A Novel Approach for Dermal Application of Pranoprofen-Loaded Lipid Nanoparticles for the Treatment of Post-Tattoo Inflammatory Reactions

**DOI:** 10.3390/pharmaceutics16050643

**Published:** 2024-05-10

**Authors:** Guillermo De Grau-Bassal, Mireia Mallandrich, Lilian Sosa, Lupe Espinoza, Ana Cristina Calpena, Núria Bozal-de Febrer, María J. Rodríguez-Lagunas, María L. Garduño-Ramírez, María Rincón

**Affiliations:** 1 Departament de Biologia, Sanitat i Medi Ambient, Facultat de Farmàcia i Ciències de l’Alimentació, Universitat de Barcelona (UB), 08028 Barcelona, Spain; gdegrau@ub.edu (G.D.G.-B.); nuriabozaldefebrer@ub.edu (N.B.-d.F.); 2 Departament de Farmàcia, Tecnologia Farmacèutica, i Fisicoquímica, Facultat de Farmàcia i Ciències de l’Alimentació, Universitat de Barcelona (UB), 08028 Barcelona, Spain; anacalpena@ub.edu; 3 Institut de Nanociència i Nanotecnologia IN2UB, University of Barcelona, 08028 Barcelona, Spain; lgarduno@uaem.mx; 4 Microbiological Research Institute (IIM), National Autonomous University of Honduras (UNAH), Tegucigalpa 11101, Honduras; lilian.sosa@unah.edu.hn; 5 Institute for Research in Applied Sciences and Technology (IICAT), National Autonomous University of Honduras (UNAH), Tegucigalpa 11101, Honduras; 6 Departamento de Química, Universidad Técnica Particular de Loja, Loja 1101608, Ecuador; lcespinoza@utpl.edu.ec; 7 Department of Biochemistry and Physiology, Faculty of Pharmacy and Food Sciences, University of Barcelona, 08028 Barcelona, Spain; mjrodriguez@ub.edu; 8 Nutrition and Food Safety Research Institute (INSA-UB), 08921 Santa Coloma de Gramenet, Spain; 9 Centro de Investigaciones Químicas, Universidad Autónoma del Estado de Morelos, 62210 Cuernavaca, Morelos, Mexico; 10 Departament de Ciència de Materials i Química Física, Facultat de Química, Universitat de Barcelona (UB), 08028 Barcelona, Spain

**Keywords:** tattoos, pranoprofen (PRA), anti-inflammatory, skin, nanostructured lipid carriers (NLC), drug delivery system, *Mus musculus* mice, hairless rats

## Abstract

Recently, the number of people acquiring tattoos has increased, with tattoos gaining significant popularity in people between 20 and 40 years old. Inflammation is a common reaction associated with tattooing. The purpose of this study was to evaluate a nanostructured lipid carrier loading pranoprofen (PRA-NLC) as a tattoo aftercare formulation to reduce the inflammation associated with tattooing. In this context, the in vitro drug release and the ex vivo permeation-through-human-skin tests using Franz cells were appraised. The tolerance of our formulation on the skin was evaluated by studying the skin’s biomechanical properties. In addition, an in vivo anti-inflammatory study was conducted on mice skin to evaluate the efficacy of the formulation applied topically after tattooing the animals. PRA-NLC showed a sustained release up to 72 h, and the amount of pranoprofen retained in the skin was found to be 33.48 µg/g/cm^2^. The formulation proved to be well tolerated; it increased stratum corneum hydration, and no signs of skin irritation were observed. Furthermore, it was demonstrated to be non-cytotoxic since the cell viability was greater than 80%. Based on these results, we concluded that PRA-NLC represents a suitable drug delivery carrier for the transdermal delivery of pranoprofen to alleviate the local skin inflammation associated with tattooing.

## 1. Introduction

Tattooing is an ancient tradition in many regions. Currently, far from being a taboo, it has become an accepted form of body art among people of all classes [1]. In some cultures, such as Polynesian tribes, tattoos are important to their religion and hierarchy. Tattoos can be useful in situations such as breast reconstruction to create the natural appearance of the nipple–areola complex, employing medical tattoos that achieve aesthetically pleasing results [2]. Similarly, tattoos may be important in forensic sciences as a possible means of identifying deceased persons, with the presence of tattoos being a secondary identifier recommended by Interpol for identifying disaster victims [3].

Recently, the number of people acquiring tattoos has increased, especially in younger generations (between 20 and 40 years old). An estimated 12% of the European population and 29% of the United States population have one or more tattoos. If this trend continues, the prevalence of tattoos among the general population will increase in the coming decades [4,5,6]. 

Despite the great popularity of this practice, there is limited information on the long-term effects of tattoos. The tattoo process bypasses the skin’s largest defense mechanism, the epidermis, which produces significant risks by exposing the body to a mixture of chemicals. The pigments contained in tattoo inks are light-stable and chemically resistant; however, they are not produced specifically for tattoos but for other industrial applications, including the manufacture of paints, textiles, or coatings, where purity requirements are not too strict. Some studies have reported that nearly 60% of tattoos worldwide are completely or partly black. The combinations of most black pigments may contain dangerous chemicals like primary aromatic amines or polycyclic aromatic hydrocarbons, which are linked to causing cancer. Carbon black has been labeled as potentially carcinogenic to humans (group 2B) by the International Agency for Research on Cancer (IARC) [7,8].

The Council of Europe’s ResAP resolution, introduced in 2003 and amended in 2008, describes specifications on the purity of tattoo inks. As of 2022, a new amendment is active that establishes concentration restrictions for 17 of the 40 compounds specifically mentioned in the ResAP Resolution that are used in tattoo inks and permanent makeup [8].

Common skin reactions after acquiring a tattoo include local inflammation with mild edema, sensitivity to touch, and sometimes pain and itching [9]. Chronic complications are mainly allergic reactions that occur especially with red tattoos. Allergic contact dermatitis is a common inflammatory skin disorder produced by exposure to non-protein chemical molecules [10,11]. Another complication is the Koebner phenomenon, which occurs in approximately 25% of patients with psoriasis, who experience the appearance of new psoriatic lesions after tattoos. Psoriasis is a chronic inflammatory disorder of the skin characterized by epidermal hyperplasia and the appearance of erythematous lesions with silvery scales [12]. Finally, granulomatous reaction with associated uveitis (TAGU) has been described in several case reports as a result of tattooing complications [13,14,15].

Inflammation is a defense response to exogenous or endogenous stimuli, including exposure to ultraviolet radiation, ionizing radiation, allergens, and pathogens, or contacts with chemical irritants (soaps, hair dyes, tattoos). During the inflammatory process, the release of pro-inflammatory cytokines and chemokines occurs to recruit inflammatory infiltrates upon detection of cell surface or intracellular perturbations [16,17]. The European standard NF EN 17169 [18], published in January 2020, recommends some options for care after acquiring a tattoo, one of which involves keeping the tattoo area covered after topical application of a specific tattoo care ointment two or three times a day for 2–3 days after. Continued hydration of the tattoo is also necessary; it is recommended to apply a non-scented moisturizing lotion for 2 or 3 weeks to prevent the tattoo from drying out [19].

Pranoprofen (PRA) is a potent non-steroidal anti-inflammatory agent (NSAID), widely used in treating inflammation and pain. PRA inhibits cyclooxygenase-1 (COX-1) and COX-2 enzymes, thus preventing the prostaglandins synthesis. PRA is often used for symptomatic treatment of ocular inflammation in the anterior segment, as well as for the acute and long-term management of rheumatoid arthritis and osteoarthritis [20]. This drug has low bioavailability and a short plasma half-life; consequently, its use is limited. Due to the systemic side effects and gastric disorders that often occur after oral administration, it is necessary to consider alternative routes of administration. Cutaneous administration offers the advantage of avoiding the hepatic first-pass effect and allows the drug to be administered directly to the area to be treated [21].

Nanotechnology allows for the manufacturing of nanosystems that deliver drugs in a controlled and sustainable manner [22]. In cutaneous delivery, these nanocarriers are a promising strategy by which to enhance drug penetration through the stratum corneum. Among various nanosystems for topical delivery of lipophilic drugs, nanostructured lipid carriers (NLCs) offer some interesting properties, including (i) the prevention of chemical degradation of drugs; (ii) the formation of a kind of drug reservoir in the skin to provide prolonged drug release; (iii) the maintenance of skin hydration; and (iv) high encapsulation efficiency of lipophilic drugs due to the presence of liquid lipids in the matrix [23].

The advantages of NLCs over other colloidal vehicles include an easy-to-use manufacturing process and high biocompatibility. NLCs consist of a blend of solid and liquid lipids. This solid structure enables the gradual release of enclosed substances and shields them from degradation, thereby enhancing the long-lasting stability of the system [24]. The cutaneous application of these nanosystems for therapeutic and cosmetic purposes has been promisingly explored in different studies [25].

In previous studies of our research group, a formulation of PRA-loaded NLC (PRA-NLC) was optimized, prepared by the high-pressure homogenization technique, and physiochemically characterized [26]. Considering that post-tattoo care with an appropriate product is essential to relieve pain, promote skin repair, and, consequently, improve the aesthetic appearance of the tattoo, the present work aimed at investigating the potential use of this lipoidal nanocarrier for the topical delivery of PRA as a tattoo aftercare formulation to reduce inflammation associated with tattooing. We considered NLC as drug carriers because these sorts of formulations are usually compatible with the skin. Additionally, they promote the penetration of the active ingredient while minimizing systemic absorption [27]. Hence, this work explored the modulation of drug release, tolerance evaluation, and assessment of the anti-inflammatory effect of the formulations after tattooing in a hairless mouse model.

## 2. Materials and Methods

### 2.1. Chemical and Reagents

Pranoprofen (2-(5H-chromeno [2,3-bpyridin-7-yl) propanoic acid) was a kind gift from Alcon Cusi (Barcelona, Spain). Tween^®^ 80 (Polyethylene glycol sorbitan monooleate) and Castor oil (*Ricinus communis* L.) were acquired from Sigma-Aldrich Química (Barcelona, Spain). Lanette^®^ 18 (stearyl alcohol) was purchased from Cognis (Dusseldorf, Germany). PEG-8 Caprylic/Capric Glycerides (LAS) was received as a gift sample from Gattefosse (Saint-Priest, France). The tattoo ink (Viking-Ink BW Black Tribal, Náquera, Spain) was purchased from Viking-Ink B&W. The MTT (3-(4,5-dimethylthiazol-2-yl)-2,5-diphenyltetrazolium bromide) utilized for cell viability was obtained from Invitrogen Alfagene^®^ (Carcavelos, Portugal), and the HaCat was obtained by Cell Lines Service (CLS, Eppelheim, Germany). MilliQ water obtained by a MilliQ^®^ Plus System lab and was used across formulations and all experiments. The phosphate buffer saline (PBS) tablets pH 7.4 were acquired from Sigma (Darmstadt, Germany) and prepared as according to the manufacturer’s instructions. All the other chemicals and reagents used in this investigation were of analytical grade.

### 2.2. Methods

#### 2.2.1. Preparation and Characterization of Nanostructured Lipid Carriers

**Figure 1:**
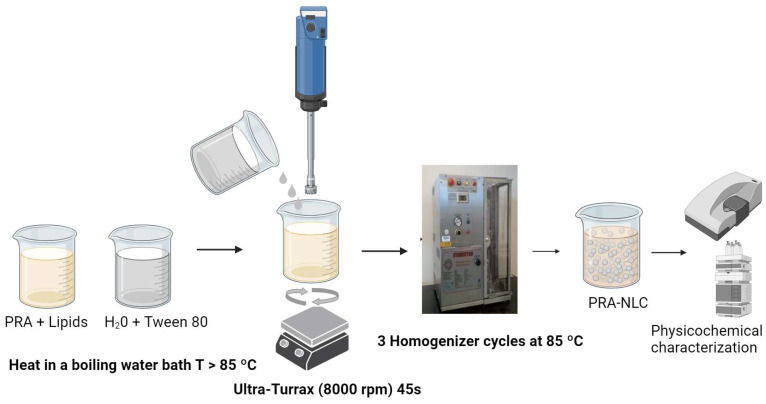
Preparation of PRA-NLC via a high-pressure homogenization technique.

PRA-loaded NLC were formulated and optimized using a 2^3^ central composite rotatable factorial design. This formulation was prepared via a high-pressure homogenization technique (Figure 1), as described previously [26,28]. The composition formula of PRA-NLC is shown in Table 1. In brief, LAS–Castor oil (75:25) as liquid lipids and Lanette^®^ 18 as the solid lipid (SL) were mixed and melted (~85 °C) in a water bath; then, the mixture was added to the melted lipid phase, and it a clear homogeneous lipid phase solution was obtained (LP). Tween^®^ 80, as a surfactant, was heated at the same temperature, and after 25 min, it was added to the lipid phase mixture, obtaining an emulsion by an Ultra-Turrax^®^ T25 (Staufen, Germany) at 8000 rpm for 45 s. The obtained emulsion was exposed to high-pressure homogenization (Homogeniser FPG 12800, Stansted, Harlow, UK) at 800 bar and 85 °C in 3 homogenization cycles, and after that, it was settled down to room temperature as the lipid recrystallized and formed the PRA-NLC suspension. Finally, the nanosuspension passed through a PVDF 0.22 µm membrane filter (Millipore Corp., Madrid, Spain) to sterilize the formulation as it was intended to be applied to tattooed—and, consequently, potentially damaged—skin. The prepared PRA-NLC batches were allowed to settle at room temperature for at least 10 h before further assays.

The prepared formulation was characterized physicochemically for mean particle size, polydispersity index, zeta potential, and encapsulation efficiency percentage [26]. The Zetasizer Nano ZS was used to determine the particle size, its distribution (polydispersity index), and the zeta potential. Measurements were performed at 25 °C on samples diluted 1:10 with MilliQ water, using disposable quartz cells for the particle size and the polydispersity index, and disposable folded capillary zeta cells for measuring the zeta potential. Results are the mean and standard deviation of three measurements.

The encapsulation efficiency (EE%) of pranoprofen in the nanoparticles was evaluated by the indirect method which consists of measuring the concentration of the drug in the medium; that is, the unencapsulated drug. The unencapsulated pranoprofen was isolated via centrifugation using Ultracel-100K centrifugal filters (Amicon Ultra, Millipore Corporation, Billerica, MA, USA) for 30 min at 4 °C at 3000 rpm (Heraeus, Multifuge 3 L-R, centrifuge, Osterode, Germany) and then analyzed via HPLC (Section 2.2.4). The EE was calculated using the following equation:

(1) $$EE(\%)=\frac{Total\;amount-Encapsulated\;amount}{Total\;amount}\times 100.$$

#### 2.2.2. In Vitro Release Assay

The in vitro release profile of PRA-NLC was determined using vertical amber glass Franz diffusion cells [29,30,31,32] (FD 400; Crown Glass, Somerville, NJ, USA) with a diffusion area of 0.64 cm^2^. A dialysis membrane (12–14 kDa), previously hydrated in methanol–water (6:4; *v*/*v*) for 24 h (Dialysis Tubing Visking, Medicell International Ltd., London, UK), was placed between the donor and the receptor compartment. Table 2 shows the experimental conditions for the in vitro release assay. The receptor fluid was phosphate-buffered saline (PBS) at pH 7.4. The system was continuously stirred at 700 rpm with magnetic beads and thermostatted at 32 ± 0.5 °C via a circulating water jacket, and sink conditions were maintained throughout the test. PRA-NLC samples of 50 µL were put into the donor compartment in direct contact with the membrane; then, aliquots of 200 µL were withdrawn from the receptor compartment at appropriate pre-established intervals of up to 3 days and replaced with an equal volume of the medium fluid (fresh PBS buffer pH 7.4). A plain solution of PRA was also tested as a control in the same conditions as PRA NLC.

The PRA content in each of the sampled aliquots was analyzed via a validated HPLC method, as described in previous studies [33]. The experimental data of the amount of PRA released were fitted to five different kinetic models—Hyperbole, first-order, Korsmeyer–Peppas, Weibull, and Higuchi (Equations (S1)–(S5) in Supplementary Material)—with GraphPad Prism^®^ (GraphPad Software Inc. version 5.0, San Diego, CA, USA) Software. The goodness of fit for each model was evaluated based on the coefficient of determination (r^2^) and the lower Akaike information criterion (AIC). 

#### 2.2.3. Ex Vivo Skin Permeation Assay

Ex vivo human skin permeation studies were carried out using vertical amber glass Franz diffusion cells (FDC 400; Crown Glass, Somerville, NJ, USA). Human skin was obtained from the surgical waste from abdominoplasty of healthy patients (Barcelona SCIAS Hospital, Spain), and it was maintained at −20 °C. The skin was dermatomed (GA630, Aesculap, Tuttlingen, Germany) at 400 µm-thick pieces, and the integrity of each skin sample was checked using a transepidermal water loss (TEWL) measurement DermaLab^®^ module (Cortex Technology, Aalborg, Denmark). Skin discs with TEWL values higher than 10 g/m^2^/h was discarded from the study and replaced by other skin discs with suitable skin integrity [34,35]. Later, these samples were fixed between the receptor and donor compartments.

**Figure 2:**
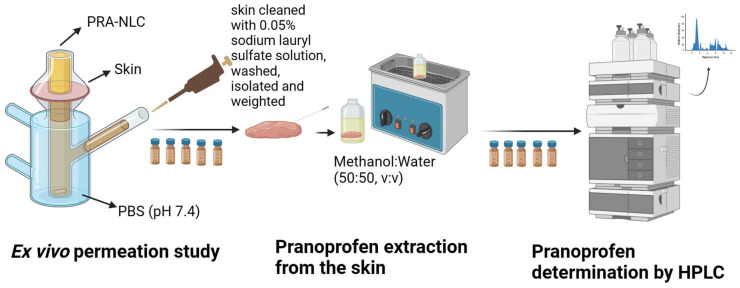
Ex vivo skin permeation study’s steps representation.

Table 3 shows the experimental conditions for the ex vivo skin permeation assay. The skin diffusion area available was 0.64 cm^2^, and the receptor compartment was filled with phosphate-buffered saline (PBS) at pH 7.4. A volume of 50 µL of PRA-NLC was added to the donor compartment in direct contact with the skin. As in the in vitro release test, a PRA solution was tested in parallel as the control. Parafilm^®^ was utilized to prevent evaporation by sealing the sampling ports and the donor compartment. Aliquots of 200 µL were withdrawn from the receptor compartment at fixed times and replaced by an equivalent volume of the receptor medium. The quantity of PRA permeated was determined via HPLC method. At the end of the permeation experiment, diffusion Franz cells were dismantled, and the skin tissues were taken out, cleaned with 0.05% sodium lauryl sulfate solution, rinsed two times with distilled water, and carefully blotted dry with filter paper [36,37]. The permeation area of the skin tissue was cut out, weighted, and perforated by a thin needle. The PRA amount retained in the skin (Q_ret_ (µg/g skin/cm^2^)) was extracted in 1 mL of methanol–water (50:50) via an ultrasound water bath technique for 15 min. Solvent samples were analyzed via HPLC, yielding the amount of pranoprofen retained in the skin. Figure 2 represents a diagram of the steps of the permeation experiment.

The steady-state flux across the skin (*Jss*, µg/h/cm^2^) was calculated by applying the following equation:

(2) Jss=QtAr×t,

where *Qt* is the quantity of drug permeated across the skin and thus detected in the receptor compartment (μg), *Ar* denotes the active cross-sectional area accessible for diffusion (cm^2^), *t* is the time of exposure (h), and the transdermal permeability coefficient (*Kp*, cm/h) was calculated by applying the following equation [38]:

(3) $$Kp=\frac{Jss}{C_{0}},$$

where *C*_0_ is the initial drug concentration in the donor compartment.

Lag time, (TL) expressed in (h), was obtained by extrapolating the regression line to the *X*-axis (X-intercept). The predicted steady-state plasma theoretical concentration of the drug (*Css*) after the topical application was calculated using the following equation:

(4) $$Css=Jss\;\cdot \;\frac{SAT}{{CL}_{P}},$$

where *Jss* is the flux, *SAT* is the hypothetical area of application (assuming an area of application of 100 cm^2^), and *CLp* is the human plasma clearance value of PRA, which, according to literature, is 609.00 cm^3^/h for older people and 1146.60 cm^3^/h, for younger people [39,40,41].

#### 2.2.4. Analytical Method for the Quantification of Pranoprofen

PRA was quantified in the samples from the drug release and permeation tests by means of high-resolution liquid chromatography (HPLC-UV), and the drug was quantified to determining the encapsulation efficiency. The method consisted of a Waters 1525 pump with a UV-vis 2487 detector (both, Waters, Milford, MA, USA). PRA was determined at 235 nm using a Kromasil 100 C-18 column (4.6 × 100 mm, 5 µm). Methanol–glacial acetic acid 5% (70:30, *v*/*v*) was the mobile phase at the flow rate of 1 mL/min. 

#### 2.2.5. In Vitro Tolerance Studies: Cytotoxicity Study by MTT Assay

The effect of different concentrations of the suspension PRA-NLC on human keratinocytes (HaCaT) was appraised using (MTT) 3-(4,5-dimethylthiazol-2-yl)-2,5-diphenyltetrazolium bromide tetrazolium cytotoxicity assay [42,43]. All these cells were obtained and cultivated in Dulbecco’s modified Eagle medium (DMEM) (2 × 10^5^ cell/mL; 96-well plate) with high glucose content buffered with 25 mM HEPES, supplemented with an extra 10% fetal bovine serum (FBS), 1% non-essential amino acids, and 1% penicillin–streptomycin at the temperature condition of 37 °C. Cells were incubated with pre-established dilutions of PRA-NLC suspension samples (from 1/10 to 1/1000) for 24 h. The HaCaT cells underwent a thorough washing with 1% sterile PBS and were then exposed to MTT solution (2.5 mg/mL) under consistent conditions for 2 h. Afterwards, the solution was removed, and 1000 μL of dimethyl sulfoxide (DMSO), a 99% pure solubilization reagent, was added to lyse the cells and dissolve the purple MTT crystals. Subsequently, the absorbance of the solution was measured at 570 nm using a LUX microplate photometer Varioskan TM (Thermo Scientific, Waltham, MA, USA). The resulting values, calculated using the provided equation, represent the percentage of cell survival relative to the negative control group (untreated HaCaT cells were used as the control with 100% viability). Cell viability was calculated according to Equation (5):

(5) $$\%\mathrm{Cell}\mathrm{viability}=\frac{\mathrm{Abs}\mathrm{sample}}{\mathrm{Abs}\mathrm{control}}\times 100.$$

#### 2.2.6. In Vivo Tolerance Studies by Monitoring Biomechanical Properties in Human Volunteers 

Changes in the skin’s biomechanical properties were monitored after the formulation application in contrast with the baseline state. In vivo skin human tolerance tests by appraising biomechanical parameters were approved by the University of Barcelona Ethics Committee on 20 March 2018 (IRB00003099). For this experiment, ten healthy-skinned participants, aged between 23 and 51, were recruited and asked to abstain from using cosmetics on the flexor side of the left forearm 12 h before the experiment. The measurement sites were delimited around 3.5 cm in diameter. The assessment of total skin water loss (TEWL) was conducted using a TEWL-Dermalab^®^ device. This device measures the quantity of water that escapes into the atmosphere surrounding the epidermal layer of the skin via processes of diffusion and evaporation [44]. The TEWL values were collected in the basal state and at different time points of 10, 30, 60, and 120 min, following the application of a thin and uniform layer of PRA-NLC. To appraise, the probe was pressed and held on the skin for 60 s in the treated area. The assessment of skin hydration levels (SCH) was carried out using a Corneometer^®^ 825 equipped with a multiprobe-installed Hydration Adapter^®^ MPA5 (Courage and Khazaka Electronics GmbH, Cologne, Germany). This device employs the capacitance method, leveraging the relatively high dielectric constant of water in comparison to other substances present in the skin. SCH values were measured prior to application in the baseline state and at 10, 30, 60, and 120 min following the application of PRA-NLC in the designated area. 

#### 2.2.7. In Vivo Anti-Inflammatory Efficacy Studies

##### Study Protocol and Animals

In vivo assays were performed in accordance with the Official Normative for Animal Care and Handling from Mexico (protocol number: NOM-062-ZOO-1999), and the Academic Ethics Committee of the Vivarium at the Universidad Autónoma del Estado de Morelos (Mexico) approved the Study Protocol on 19 January 2023. The anti-inflammatory potential of PRA-NLC was tested using two in vivo models: the first of them corresponded to a xylol-induced acute inflammation experiment using a *Mus Musculus* mouse ear model; while the second assay was carried out after to produce a tattoo in a hairless rat model.

##### Xylol-Induced Inflammation Model in Mouse Ear

For the xylol-induced edema model in mice, 10-week-old male *Mus Musculus* mice were utilized. The mice were adapted over 7 days to a 12:12 h light–dark cycle with ad libitum access to water and food. Temperature and humidity were kept controlled. The mice were divided into three groups. The inflammatory process was induced topically by applying xylol to the left ear [45]. After 25 min of xylol application, one group (*n* = 6) was treated with PBS (positive control), whereas a second group was treated with PRA-NLC (*n* = 6). In addition, a third group of healthy and non-treated mice was used as a negative control (*n* = 6). Mouse ear thickness was evaluated before and after inducing inflammation, as well as 20 min after the treatment with PRA-NLC or PBS, respectively. The ear thickness was measured via a digital thickness gauge (from 0 to 10 mm) (Mitutoyo Corp, Kawasaki, Japan). Edema was assessed by measuring the variance between the initial ear thickness and the thickness after application of xylol. The anti-inflammatory efficacy was quantified by the reduction in edema following treatment with PRA-NLC. Upon completion of the study, the mice were euthanized via cervical dislocation, and histological assays were conducted on the excised left ears.

##### Post-Tattoo Inflammation Model on the Back in Hairless Rats

**Figure 3:**
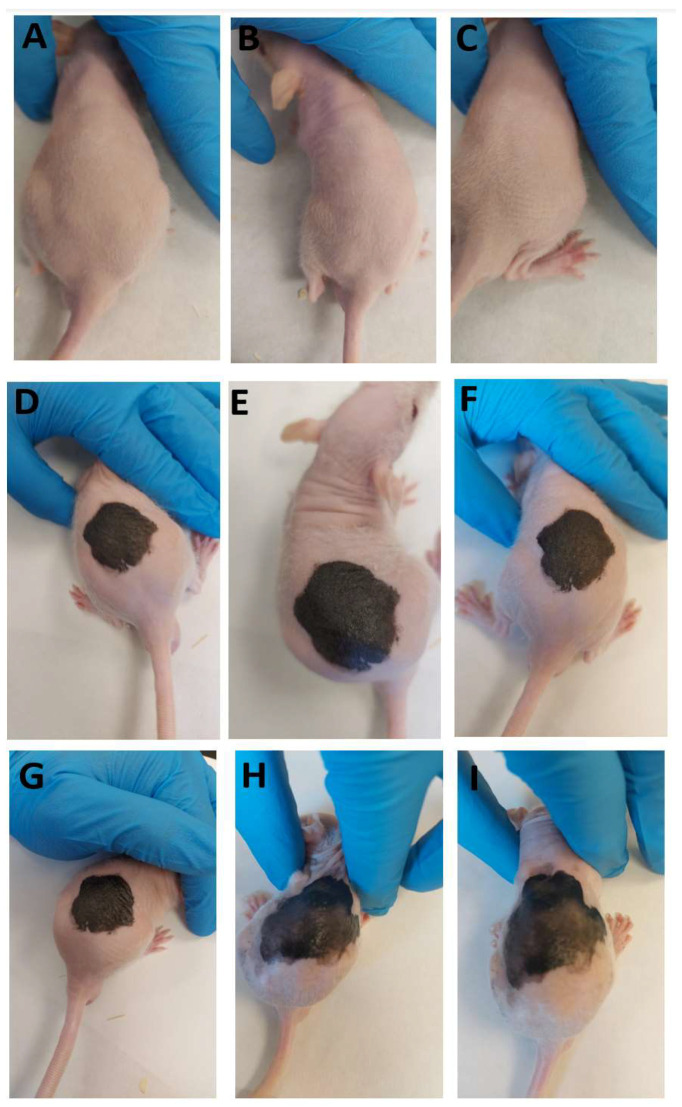
Experimental back tattoo groups in a hairless mouse model: (**A**–**C**) negative control at 5, 25, and 45 min, respectively; (**D**–**F**) positive group at 5, 25, and 45 min, respectively; (**G**–**I**) group treated with PRA-NLC at 5, 25, and 45 min, respectively.

This experiment was carried out using 10-week-old hairless female rats. Every effort was made to minimize the number of rats used in the experiment and minimize animal suffering. The animals were allowed to adapt for 5 days under controlled temperature and humidity; then, they were tattooed using a 0.5 mm tattoo needle to tattoo black ink delicately and superficially into a delimited skin area. Additionally, we used a glove to distribute the ink into the exposed skin area. Six rats were tattooed as a group to form a positive control (tattooed with no pharmacological treatment). A second group (*n* = 6) was tattooed and then treated with PRA-NLC. And a final group of healthy rats served as a negative control; the animals were not tattooed and did not receive any pharmacological treatment either (*n* = 6). Figure 3 shows photos of the backs of the different experiment groups after 5, 25, and 45 min of assay. 

**Figure 4:**
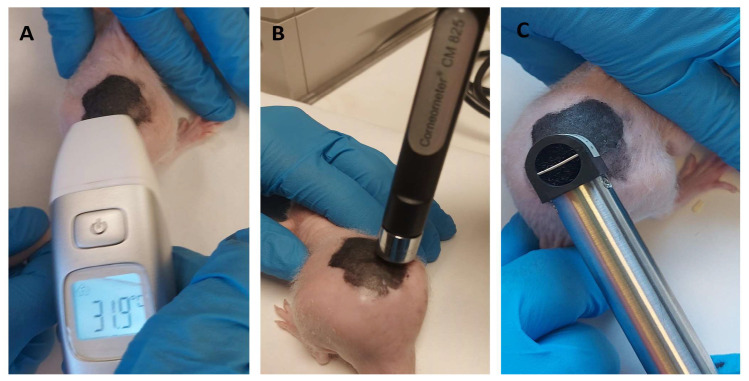
Devices for the measure of biochemical parameters in the backs of hairless rats: (**A**) thermometer to measure T (°C); (**B**) Corneometer^®^ probe to measure SCH (arbitrary units); (**C**) TEWL-Dermalab^®^ probe to measure TEWL (g/h·m^2^).

The following biomechanical properties were evaluated on the rats’ back skin: SCH, TEWL, and temperature before the inflammation (baseline time point), after making the tattoos, and after treating the animals of group II with PRA-NLC. The measurements were made at the same time points for the three groups. TEWL was measured using a TEWL-Dermalab^®^, SCH was evaluated using a CM-825 Corneometer (Courage and Khazaka Electronics GmbH, Köln, Germany), and cutaneous temperature was measured using a digital thermometer. Rats were euthanized after finishing the study, and the backs were cut to carry out histological assays. Figure 4 shows images of the devices used for the measure of biochemical parameters in the skin of hairless rats.

##### Histological Studies

Histological analysis was carried out for the two in vivo experiments. The samples of the left ears from *Mus Musculus* mice and back skin from hairless rats were suspended in PBS for 3 h (replacing it with fresh medium in time intervals of 1 h). Then, they were preserved in a 4% buffered formaldehyde solution for 24 h, dehydrated in gradient ethanol, and then embedded in melted paraffin. The resulting blocks were cut into 5 mm sections, stained with hematoxylin and eosin, and observed using a microscope (Olympus BX41, Hamburg, Germany) equipped with an Olympus XC50 camera. Images were obtained at 100× magnification.

## 3. Results

### 3.1. Physicochemical Characterization of the PRA-NLC

PRA-NLC exhibited a homogeneous appearance without the presence of visible precipitates. This formulation showed a mean particle size of around 220 nm, a negative surface charge with zeta potential values around −11 mV, and polydispersity index values lower than 0.25. The encapsulation efficiency percentage shows values around 98% of PRA in the lipid core (Table 4). 

### 3.2. In Vitro Active Pharmaceutical Ingredient-Release Kinetics Studies

**Figure 5:**
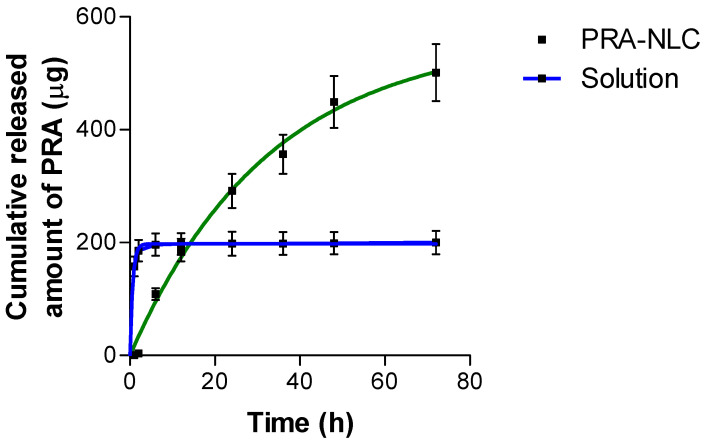
In vitro release profile of PRA from the nanostructured lipid carriers compared to the plain solution.

The ability of PRA-NLC to release the incorporated drug was tested via a diffusion study using Franz diffusion cells, and the kinetic model that best fits the experimental data was chosen based on the coefficient of determination (r^2^) closest to 1, the lowest value of the AIC, and the simplest model [32,46]. The drug release process was greater during the first 24 h for the nanostructured formulation; PRA was shown to be completely available from the solution in just 3 h. At the end of the experiment, PRA-NLC was able to release ~70% of the drug content. The PRA release profile from the nanostructured lipid carrier followed a first-order kinetic model, while the plain solution profile was best described by the Weibull model (Figure 5). Table 5 reports the results of the kinetic modeling (full data are given in Tables S1 and S2). 

### 3.3. Ex Vivo Skin Permeation Studies

Ex vivo permeation studies were carried out in Franz diffusion cells, using human skin as the permeation membrane. The results of the permeation parameters—flux (*J_ss_*, μg/h/cm^2^), permeability coefficient (*Kp*, cm/h), lag time (TL, h), the cumulative amount of PRA permeated after 24 h of experiment (Q_24h_ µg), and the amount retained in the skin (Q_ret_ µg/g/cm^2^)—are described in Table 6. 

Table 7 shows the predicted steady-state plasma concentrations (C_ss_, μg/mL), the values of which were below the reported therapeutic plasma concentration (4.89 ± 1.29 µg/mL for older humans and 10.19 ± 2.43 µg/mL for younger humans) [39,40,41]. These results suggest that the dermal application of PRA-NLC may not have systemic effects, which guarantees a safe local anti-inflammatory and analgesic effect of the PRA.

### 3.4. Cytotoxicity Study by MTT Assay

**Figure 6:**
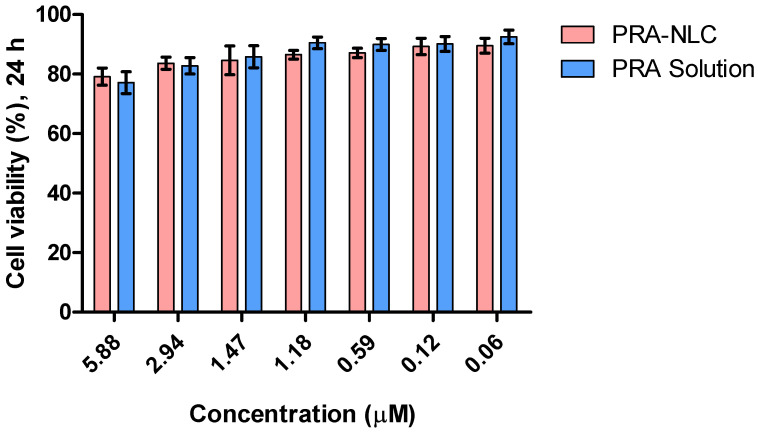
In vitro cytotoxicity of PRA-NLC suspension and the plain solution of PRA in HaCaT cells exposed for 24 h at different concentrations.

This test evaluates cell viability, defined as a parameter that measures the overall activity of a cell population as the ability of cellular mitochondrial dehydrogenases to reduce the vital dye MTT, which correlates with the total number and/or vitality of living cells, depending on the endpoint measured and the test design used [47]. After 1 day of incubation, it was observed that the tested dilutions of the formulation (1/10 to 1/1000) did not affect cell viability in nearly 90% of the examples, i.e., in six of the seven dilutions illustrated in Figure 6. 

### 3.5. In Vivo Tolerance Studies by Monitoring Biomechanical Properties in Human Volunteers

**Figure 7:**
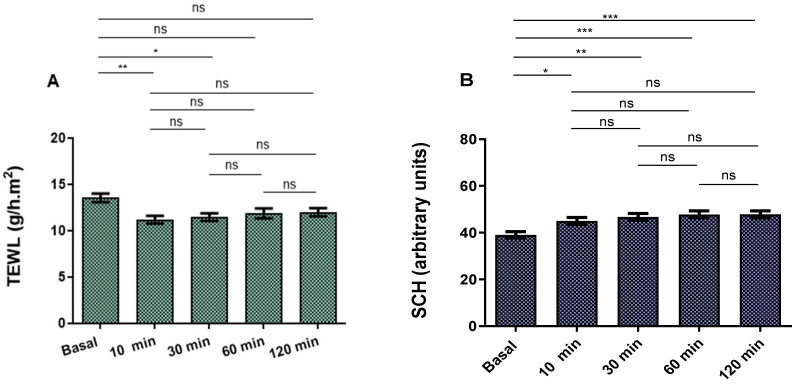
Skin biomechanical parameters on healthy-skin volunteers: (**A**) TEWL (g/h·m^2^) of PRA-NLC; (**B**) stratum corneum hydration of PRA-NLC expressed as arbitrary units (*n* = 10). *p*-values: * *p* < 0.05, ** *p* < 0.01, *** *p* < 0.001. ns = non-significant.

The evolution of transepidermal water loss (TEWL) and stratum corneum hydration (SCH) parameters at basal state and after applying PRA-NLC after 10, 30, 60, and 120 min are shown in Figure 7. A little but not statistically significant decrease in the TEWL values of the NLCs was recorded, demonstrating an occlusive effect without changes in skin integrity. Despite this, there was a slight but statistically significant increase in SCH. Given the fact that skin capacitance is strongly associated with skin hydration, these results indicated that PRA-NLC raised to some extent the hydration associated with normal skin behavior. No visible signs of skin irritation were observed as a consequence of the formulation application on the surface of the skin of the research participants, indicating that PRA-NLC was well tolerated on the skin.

### 3.6. Anti-Inflammatory Efficacy Studies

#### 3.6.1. Xylol-Induced Inflammation Model in Mouse Ear

**Figure 8:**
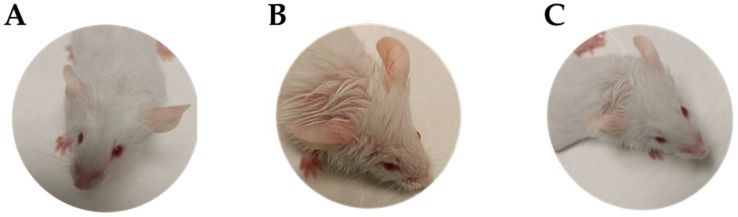
Macroscopic appearance of the ear’s aspect: (**A**) negative control group (control condition); (**B**) positive control group (redness and edema); (**C**) PRA-NLC treatment group.

Macroscopic features of the ear’s aspect are shown in Figure 8. Topical applications of xylol on the ear of mice caused visible erythema and edema after 10 min. PRA-NLC improved these symptoms after 20 min of treatment.

**Figure 9:**
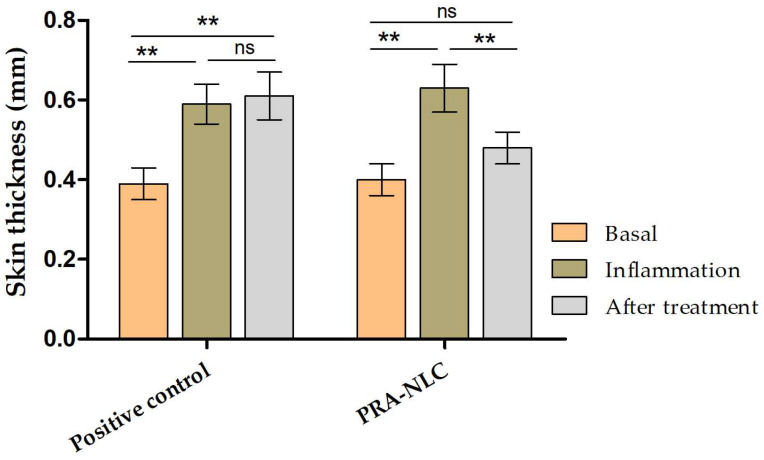
Skin thickness values of the mouse ears (mm). *p*-values: ** *p* < 0.01. ns = non-significant.

Figure 9 shows that the topical application of xylol caused edema in the ears of the mice, which was evidenced by a significant increase in their thickness after 25 min of application. However, the topical treatment with PRA-NLC significantly decreased this inflammatory symptom after 20 min of treatment, whereas the positive control only treated with PBS did not show a decrease in the inflammatory process.

**Figure 10:**
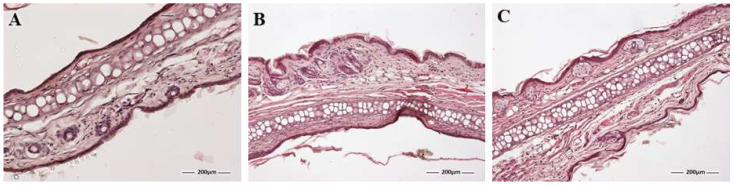
Representative images of histological sections obtained from xylol-induced inflammation model: (**A**) control conditions; (**B**) positive control; (**C**) PRA-NLC group. The asterisk indicates leucocyte infiltrate. e = epidermis; d = dermis; ac = auricular cartilage 100× magnification. Scale bar = 200 µm.

Histological analysis of mouse ear samples (Figure 10) showed that topical application of xylol caused mild infiltration of white blood cells (Figure 10B). However, when treated with PRA-NLC (Figure 10C), the animals did not display this leukocyte infiltration, indicating that the inflammation was prevented.

#### 3.6.2. Post-Tattoo Inflammation Model on the Back in Hairless Rats

**Figure 11:**
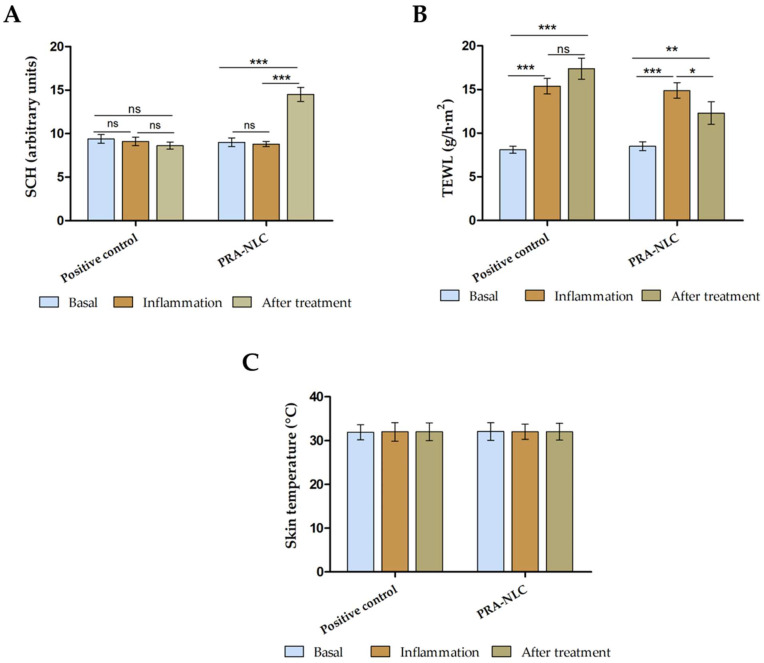
Biomechanical properties evaluation from the back-tattoo-in-hairless-rat experiment: (**A**) stratum corneum hydration (SCH); (**B**) transepidermal water loss (TEWL); (**C**) skin temperature. *p*-values: * *p* < 0.05, ** *p* < 0.01, *** *p* < 0.001, ns = non-significant.

Tattooing caused a slight-but-not-significant reduction of SCH after 25 min. However, the effect of PRA-NLC on SCH after 20 of treatment was remarkable, indicating their hydration potential (Figure 11A). The hydration level is a function of the water concentration gradient between the dermis and the skin’s surface. An increase in skin water corresponds to an augmentation in the permeability of topically applied compounds. Figure 11B shows an increase in the value of transepidermal water loss (TEWL) after 25 min of tattooing, which confirms damage to the integrity of the skin. However, topical application of PRA-NLC significantly restored this parameter after 20 min of treatment compared with the positive control, whose value continued to increase over time. Regarding temperature variation, the change was barely appreciable for temperature values in the case of rat skins (Figure 11C).

**Figure 12:**
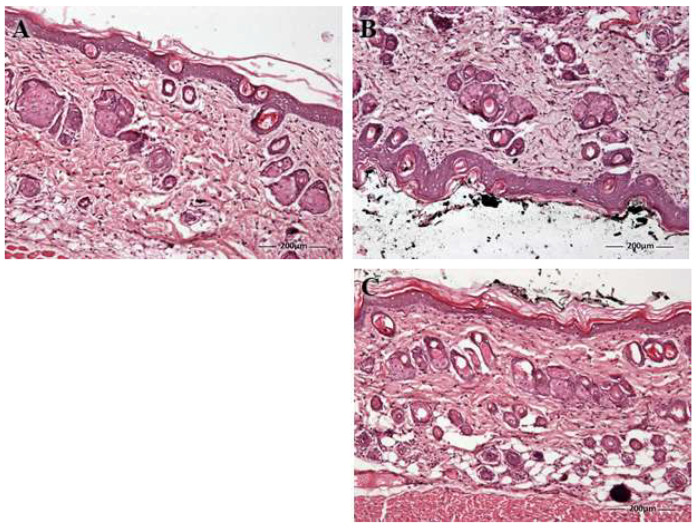
Histological images of skin sections obtained from the back-tattoo-in-hairless-rat experiment: (**A**) negative group or control conditions; (**B**) positive group: tattooed skin and covered with ink; (**C**) PRA-NLC group, i.e., treatment with PRA-NLC after making a tattoo and covering with ink. Asterisk indicates increased epithelium. e = epidermis; d = dermis; sc = stratum corneum 100× magnification. Scale bar = 200 µm.

Finally, histological evaluation shows the normal conditions of the epidermis and dermis observed in the negative control group (Figure 12A). The epidermis is the outermost layer and consists of several layers of cells, while the dermis is a thicker layer that contains connective tissue, blood vessels, and nerve endings. Normally, tattooing harms the topmost layer of the skin, including the epidermis, epidermal–dermal junction, and the papillary layer of the dermis. Figure 12B showed an increase in the epithelium in the positive control as a result of the inflammation caused by the tattoo. PRA-NLC reversed this increase, showing its anti-inflammatory potential (Figure 12C).

## 4. Discussion

The aftercare in tattooing commonly includes washing the area twice or thrice a day and apply moisturizers several times. However, feeling some pain, discomfort, and inflamed skin is also frequent because the skin has been pierced several times to insert the ink into the skin, and it may be necessary to use over-the-counter medications with an anti-inflammatory drug to alleviate pain and inflammation. The clinical needs in tattooing encompass infection prevention, pain management, identifying pre-existing skin conditions and potential allergies or sensitivities to tattoo inks, promoting proper healing, and minimizing the risk of complications, such as infection, scarring, or color fading. Patients expect detailed instructions on how to care for their new tattoo, including guidelines for cleaning, moisturizing, and protecting the tattooed area to promote optimal healing. This involves advice on using appropriate skincare products that facilitate prompt healing and recovery of the tattooed area while preserving the quality and vibrancy of the tattoo over time. In the present study, a PRA-loaded nanostructured lipid carrier for topical application was designed to modulate the drug release and promote its retention in human skin to offer a safe and effective alternative treatment for inflammatory events after tattooing. 

For PRA-NLC formulation, excipients designated as “generally recognized as safe” (GRASS) by the United States Food and Drug Administration (FDA) were used. Castor oil and PEG-8 caprylic/capric glycerides (LAS) were used as the liquid. Castor oil is a natural non-volatile oil of high viscosity, pale yellow in color, and whose main component is a ricinoleic acid: glyceride. Castor oil is used in dermal formulations due to its ability to promote the drug’s permeation through the skin [48,49]. LAS is used as a non-ionic and non-toxic excipient in dermal products such as creams or lotions. In micro- and nanoemulsions, this excipient has been explored as a surfactant to form a film around the nanodroplets, producing a decrease in the interfacial tension and smaller droplet size [50]. On the other hand, the solid lipid ingredient used in the final formulation was Lanette^®^ 18 (Stearyl alcohol), which is used in cosmetics and topical pharmaceutical creams and ointments as a stiffening agent [51]. Finally, the oleate ester of sorbitol “Tween^®^ 80” was used as the surfactant. This excipient is a water-soluble and non-ionic synthetic product with an HLB of 15 [52]. PRA-NLC was prepared by applying high-pressure homogenization technology, with which, through different forces, including hydraulics, cavitation, and turbulence, it is possible to achieve a small particle size [53]. This method offers a simple industrial scale-up process, which can be applied several times to obtain the desired droplet size [53,54]. 

The characterization results confirmed that the composition formulation of PRA-NLC and the preparation method were appropriate and robust. The small particle size (220.402 nm) could enhance the adhesiveness with the tissues and provide closer contact between the nanosystem and the skin [55]. The obtained polydispersity index value (PI) of 0.241 is within the optimal range for this type of formulation (0.1 to 0.25), showing a narrow size distribution and suggesting long-term physical stability [56]. PRA-NLC also showed a high encapsulation efficiency (98%). 

The release profile is used as an important tool to evaluate drug availability and its diffusion rate from formulations [57]. Drug release is affected by morphometric/morphological parameters, the class of surfactant, and the preparation method [58]. Different kinetic models were compared, such as Hyperbole, first-order, Korsmeyer–Peppas, Weibull, and Higuchi, to select the one that best described the release kinetics [59]. PRA released from the nanostructure lipid carrier followed a first-order kinetic model with a constant (Kf) of 0.031 ± 0.005 h^−1^; the constant corresponds to the release rate at which the drug is released from the formulation, and higher Kf values represent faster drug release. The first-order model assumes that the release rate is directly correlated to the amount of drug remaining in the formulation; that is, the drug release rate decreases over time [60,61]. This profile can be seen in Figure 5, in which after 48 h, the diffusion of the drug through the dialysis membrane was minimal. These results suggest that PRA-NLC releases the drug in a sustained manner, and it possesses the kinetic profile characteristic of prolonged drug release formulations. In contrast, PRA solution showed fast drug release. Weibull was the model that best described the release profile; β is the shape parameter and, depending on its value, the profile can fit either exponential drug release (β = 1), a sigmoid S-shape (β > 1), or parabolic drug release consistent with exponential drug release (β < 1) [62]. The release exponent (n) in the Korsmeyer–Peppas model permits the investigation of the mechanism that governs the drug release (Tables S1 and S2). When the value of n falls within the range of 0.45 to 0.89, as is the case for PRA-NLC (*n* = 0.63), drug release is considered to exhibit the non-Fickian transport of spherical systems, suggesting that the release is driven by diffusion [63,64]. Differently, the *n* value for PRA-solution (Table S2) was 0.04, indicating that the drug was released according to a Fickian diffusion (*n* < 0.45) [65]. 

Investigating the permeation of a drug through ex vivo skin is necessary for developing and optimizing drug delivery systems intended for topical use. The ex vivo permeation studies provide valuable data to predict drug behaviour. The permeation capacity of a drug depends on the physicochemical properties of the drug [66], as well as the formulation and the affinity of the drug for the tissue. During the ex vivo permeation study, different aliquots of receptor medium were collected at different time intervals for a maximum of 24 h. At the end of the experiment, PRA was extracted from the skin to evaluate the amount of drug that penetrates through the skin and remains inside it [67,68]. Concentrations of all samples were obtained, and cumulative amounts of drug permeation were plotted, expressed as the median (min–max). Least squares regression was performed from the linear zone of the plot in order to calculate the skin permeation parameters. The results showed that PRA permeates through the skin with a flux (*J_ss_*) of 0.1598 µg h^−1^ cm^−2^ for PRA-NLC, which was about four-fold higher than the flux obtained for PRA solution. The flux represents the permeation rate through the skin once the steady state has been achieved. The permeability coefficient (*K_p_*) indicates the ability of the drug in diffusing through the skin; in this study, the *K_p_* value was 1.07 × 10^−5^ cm h^−1^. According to the lag time (TL), PRA would reach a steady state in 5.7 h when delivered from NLC, and 4.1 h from the solution. The amount of PRA that permeated within 24 h (Q_24h_) was 2.14 µg for PRA-NLC, while it was a quarter of that for the PRA solution. Concerning the retained drug amount in the skin (Q_ret_), the results showed a high retention value of 33.482 µg g^−1^ cm^−2^ for PRA-NLC and slightly lower (20.50 µg g^−1^ cm^−2^) for the PRA solution, indicating that the drug is capable of passing through the stratum corneum and remaining within the deepest layers of the skin, reaching an effective drug concentration in the target area and achieving a prolonged anti-inflammatory effect thanks to the slow release of the drug. This investigation discloses sufficient evidence that targeting and a prolonged effect can be accomplished with immense perspective in dermal delivery. Regarding the results obtained from the predicted steady-state plasma concentrations of PRA (*Css*) for both PRA-NLC and the PRA solution, assuming an area of application of 100 cm^2^ of skin, they were far below the reported therapeutic plasma concentration achieved through oral administration (4.89 ± 1.29 µg/mL and 10.19 ± 2.43 µg/mL for older and younger humans, respectively) [39,40,41]. These values guarantee that the dermal application of the nanosuspension would not have any systemic effect; thus, they guarantee a safe local anti-inflammatory and analgesic effect. 

The tolerance of PRA-NLC was assessed via in vitro and in vivo experiments. In accordance with OECD guidelines, a substance is considered irritating or cytotoxic when it causes a mean tissue viability of less than 50% compared to a negative control after 15 to 60 min of exposure. In this study, the results of the in vitro cytotoxicity assay suggested that the dilutions of PRA-NLC tested did not cause damage to the HaCaT cells, which showed a viability greater than 80%, indicating that the developed formulation is non-irritant and could be used on the skin [47,69]. These findings were supported by in vivo studies by evaluating skin biomechanical properties such as transepidermal water loss (TEWL) and stratum corneum hydration (SCH) after applying PRA-NLC on the flexor side of the forearm in ten healthy subjects (Figure 7). TEWL is an indirect measure of skin barrier function and integrity by indicating the level of water evaporation rate from the skin surface [70,71]. This parameter is correlated with SCH such that TEWL increases while SCH decreases when the skin barrier is damaged by conditions such as cuts, burns, or certain skin conditions, including psoriasis, rosacea, and dermatitis. The results of this study showed that TEWL values increased after causing inflammation and decreased at 120 min after application of PRA-NLC, while SCH increased. These findings indicate that PRA-NLC does not modify or harm the skin surface; instead, it functions as an occlusive humectant, which helps to prevent transepidermal water loss, consequently enhancing skin hydration. This effect is likely attributed to the lipid composition of the nanocarriers [27]. 

The efficacy of PRA-NLC was analyzed using two in vivo models: a xylol-induced acute inflammation model in mouse ears and a post-tattoo inflammation model on the back in hairless rats. The inflammatory pathway is a complex pathophysiological response that helps the body defend itself against pathogens that cause damage at the cellular level. The inflammatory process at the skin level manifests itself with various signs and symptoms, including increased skin thickness, erythema, dry skin, infiltration of inflammatory cells, and the release of various inflammatory mediators. Increased skin thickness is indicative of edema, increased vascular permeability, and keratinocyte proliferation [72,73]. In this study, the topical application of xylol caused an increase in skin thickness due to the inflammatory reaction. On the contrary, topical treatment with PRA-NLC decreased this parameter after 20 min of treatment, showing no significant differences with the initial state (Figure 9). Finally, the post-tattoo inflammation model in the hairless rat back revealed that the integrity of the skin was compromised by significantly increasing the TEWL after the tattoo was performed. This result could be due to a destabilization of the skin surface and the follicular ducts, causing a modification in the stratum corneum [74,75]. However, topical treatment with PRA-NLC significantly restored this parameter while increasing SCH, demonstrating its hydration potential after 20 min of treatment. Histological evaluation showed damage to the stratum corneum, slight edema, and leukocyte infiltration in the tattooed skin of the positive control. All of these symptoms were reversed after treatment with PRA-NLC, supporting the efficacy of the formulation in the treatment of post-tattoo inflammation.

Although results from the tolerance study and the in vivo efficacy study in mice suggest that the use of the formulation as tattoo aftercare is safe, it is not without risk. Besides inflammation, the skin may become infected if not properly cared for; for this reason, our formulation was sterilized via filtration to prevent skin infection when applying the product to the tattooed area. Our investigation covered the evaluation of short-term product application just a few days after tattooing to alleviate the pain associated with the inflammation caused by the tattoo. Although our formulation is not intended to be used for long periods, the long-term effect of using the product should also be investigated in future studies.

It is worth mentioning that in this work, the tolerance study was conducted on subjects with healthy skin, and the in vivo study in mice was conducted on healthy animals. However, the reality is that tattooing is not limited to people with healthy skin. Kluger et al. reviewed the motivations of people acquiring a tattoo, discussing the risks and recommendations of tattooing in patients with different skin conditions, such as sensitive skin, atopic dermatitis, psoriasis, or even diabetic skin, among others [76]. Hence, one limitation of this study is its considering only healthy skin. This approach is useful in the early research stages and preclinical phases to evaluate the formulation performance. Nonetheless, the use of our formulation should also be evaluated in future clinical studies addressing sensitive skin and other skin conditions.

## 5. Conclusions

In summary, the findings indicate that the formulated PRA-NLC suspension could serve as an efficient system for delivering and controlling the release of PRA into the skin. PRA-NLC enhanced the drug’s biopharmaceutical profile, with an approximately four-fold increase in the flux and improved drug retention within the skin, and consequently, this may enhance the drug’s local anti-inflammatory and analgesic effects. The nanosuspension showed suitable physicochemical properties for topical delivery, with a mean particle size of about 220 nm, high encapsulation efficiency (≈98%), and sustained drug release over 72 h. Because the formulation is intended to be applied to pierced skin due to the tattoo, the sterilized formulation could be applied in a spray dosage form to prevent infection. Additionally, it was well tolerated, as demonstrated by the slight decrease in TEWL values on the volunteers’ forearm (from 13.5 to 12 g/h·m^2^), and no irritation was observed during the study. Additionally, the formulation showed a moisturizing effect (SCH values increased from about 40 AU to 50 AU). The formulation also exhibited a good anti-inflammatory profile, as evidenced by the approximately 35% decrease in the mice’s ear thickness in relation to the positive control. This formulation is thus a promising candidate for offering, in clinical application, local anti-inflammatory activity after tattooing. In this regard, future pre-clinical and clinical research might be needed to confirm the effectiveness and safety of the product in the clinical setting. Moreover, the long-term effects of using the formulation should be considered, and its use in sensitive skin and other skin conditions should also be addressed in future research.

## Figures and Tables

**Table 1:** Composition of pranoprofen’s nanostructured lipid carriers (PRA-NLC).

Composition of PRA-NLC					
cPRA ^1^ (%)	cTW80 ^2^ (%)	cSL/LP ^3^ (%)			Milli-Q Water (%)
		Lanette^®^ 18 (SL)	LAS (LL)	Castor Oil (LL)	
1.50	2.50	2.50	1.88	0.63	91.00

^1^ (cPRA) concentration of PRA; ^2^ (cTW80) concentration of the surfactant Tween^®^ 80; ^3^ lipid phase (LP) (which is 5%, referring to the total formulation); SL: solid lipid; LL: liquid lipids.

**Table 2:** Experimental conditions for the in vitro release assay.

Condition	Specification
Receptor fluid	Phosphate buffered saline (PBS pH = 7.4)
Cell volume (mL)	5
Membrane	Dialysis membrane
Active diffusion area (cm^2^)	0.64
Temperature (°C)	32 ± 0.5
Stirring speed (r.p.m.)	700
Dose (µg)	750
Sample volume (µL)	200
Sampling point times (h)	1, 2, 6, 12, 24, 36, 48, 72
Number of replicates	*n* = 5

**Table 3:** Summary of the experimental conditions for the ex vivo skin permeation assay.

Condition	Specification
Receptor fluid	Phosphate buffered saline (PBS pH = 7.4)
Cell volume (mL)	4
Membrane material	Abdominal human skin
Active diffusion area (cm^2^)	0.64
The thickness of the skin (µm)	400
Temperature (°C)	32 ± 0.5
Stirring speed (r.p.m.)	700
Dose (µg)	750
Sample volume (µL)	200
Sampling point times (h)	6, 10, 12, 16, 19 and 24.
Number of replicates	*n* = 5

**Table 4:** Physicochemical characterization of pranoprofen-loaded nanostructured lipid carriers (PRA-NLC).

Physicochemical Characterization			
Mean Particle Size (nm) ± SD *	Polydispersity Index ± SD *	Zeta Potential (mV) ± SD *	Encapsulation Efficiency (%) ± SD *
220.40 ± 8.36	0.24 ± 0.05	−11.07 ± 0.32	98.06 ± 0.09

* SD = standard deviation (*n* = 3).

**Table 5:** Kinetic modeling for PRA released from the nanostructured formulation and the plain solution according to the best fit (first-order model). Amax: maximum amount predicted by the model; Kf: drug release rate; β: shape factor and td: time necessary to release the 63.2% of the drug.

	Kinetic Model	Amax	Kf	β	td
PRA-NLC	First-order	565.30 ± 34.52	0.03 ± 0.00	-	-
PRA-solution	Weibull	1.98 ± 3.86	-	0.79 ± 0.44	0.56 ± 0.24

**Table 6:** Skin permeation parameters of PRA calculated from experimental data obtained from ex vivo permeation study. Results are described as the median (minimum–maximum range values) (*n* = 5).

		Biopharmaceutical Parameters			
	*J_ss_* (µg/h/cm^2^)	*K_p_* (×10^5^ cm/h)	TL (h)	Q_24h_ (µg)	Q_ret_ (µg/g/cm^2^)
PRA-NLC	0.1598 (0.1434–0.1861)	1.07 (0.96–1.24)	5.73 (2.91–8.72)	2.14 (1.54–2.31)	33.48 (31.41–34.00)
PRA-solution	0.0388 (0.0368–0.0393)	2.99 (2.83–3.02)	4.10 (4.01–4.17)	0.49 (0.35–0.81)	20.50 (5.79–71.47)

**Table 7:** Predicted steady-state plasma concentration (C_ss_) of the PRA, assuming an area of application of 100 cm^2^ of skin. Results are described as the median, minimum, and maximum range values (*n* = 5).

	Younger Humans Css (ng/mL)	Older Humans Css (ng/mL)
PRA-NLC	13.94 (12.51–16.23)	26.25 (23.55–30.56)
PRA-solution	3.39 (3.21–3.43)	6.38 (6.04–6.45)

## Data Availability

The data presented in this study are available in this article (and Supplementary Material).

## Supplementary Materials

The following supporting information can be downloaded at https://www.mdpi.com/article/10.3390/pharmaceutics16050643/s1: Table S1: Kinetic modeling for PRA released from the nanostructured formulation. Amax: maximum amount predicted by the model; Kd, Kt, Kk, Kh: drug release rate; n: drug release mechanism; β: shape factor and td: time necessary to release the 63.2% of the drug; Table S2: Kinetic modeling for PRA from the plain solution. Amax: maximum amount predicted by the model; Kd, Kt, Kk, Kh: drug release rate; n: drug release mechanism; β: shape factor and td: time necessary to release the 63.2% of the drug; Equation (S1): Hyperbole model; Equation (S2): First-order model; Equation (S3): Korsmeyer-Peppas; Equation (S4): Weibull model; and Equation (S5): Higuchi model.
