# Supplementary material for: A Novel Approach for Dermal Application of Pranoprofen-Loaded Lipid Nanoparticles for the Treatment of Post-Tattoo Inflammatory Reactions
Source: Pharmaceutics. 2024 May 10;16(5):643. doi: 10.3390/pharmaceutics16050643 (PMC11125123; doi:10.3390/pharmaceutics16050643)
Supplement: Supplementary file 1 [file pharmaceutics-16-00643-s001.zip › pharmaceutics-2884230-supplementary.pdf]

pharmaceutics

Supplementary Materials

A Novel Approach for Dermal Application of Pranoprofen-Loaded Lipid Nanoparticles for the Treatment of Post-Tattoo Inflammatory Reactions

Guillermo De Grau-Bassal [1,†], Mireia Mallandrich [2,3,*,†], Lilian Sosa [4,5], Lupe Espinoza [6], Ana Cristina Calpena [2,3], Núria Bozal-de Febrer [1], María J. Rodríguez-Lagunas [7,8], María L. Garduño-Ramírez [3,9] and María Rincón [3,10,*]

Tables S1 and S2 show the results of the kinetic modelling for the NLC-PRA and the plain solution, respectively. The best-fit model was selected based on the Akaike's Information Criterion (AIC), the lower AIC, the better the fit. For both formulations, the NLC-PRA and the PRA plain solution the first-order kinetic model was the one which best described the drug release process.

Table S1. Kinetic modelling for PRA released from the nanostructured formulation. Amax: maximum amount predicted by the model; Kd, Kt, Kk, Kh: drug release rate; n: drug release mechanism; β: shape factor and td: time necessary to release the 63.2 % of the drug.

**Kinetic Model**	**Parameters**	**Units**	**Value (Mean ± SD)**
Hyperbole	Kd	h	42.3 ± 7.4
	Q∞	μg	807.4 ± 70.9
	R^2	-	0.9735
	**AIC**	-	**93.66**
First-order	Kf	h^{-1}	0.0305 ± 0.0038
	Q∞	μg	565.3 ± 34.5
	R^2	-	0.9736
	AIC	-	93.60
Korsmeyer-Peppas	Kk	h^{-n}	36.7 ± 6.3
	n	-	0.6273 ± 0.0463
	R^2	-	0.9547
	AIC	-	98.47
Weibull	td	h	32.3 ± 6.9
	β	-	1.01 ± 0.12
	Q∞	μg	561.0 ± 58.1
	R^2	-	0.9736
	AIC	-	95.60
Higuchi	Kh	h^{-1}	58.9 ± 1.9
	R^2	-	0.9329
	AIC	-	100.01

Table S2. Kinetic modelling for PRA from the plain solution. Amax: maximum amount predicted by the model; Kd, Kt, Kk, Kh: drug release rate; n: drug release mechanism; β: shape factor and td: time necessary to release the 63.2 % of the drug.

Kinetic Model	Parameters	Units	Value (Mean ± SD)
Hyperbole	Kd	h	0.2434 ± 0.0718
	Q∞	μg	201.4 ± 4.2
	R^2	-	0.9735
	AIC	-	82.94
First-order	Kf	h^{-1}	1.558 ± 0.221
	Q∞	μg	197.9 ± 3.6
	R^2	-	0.9421
	**AIC**	-	**82.71**
Korsmeyer-Peppas	Kk	h^{-n}	173.3 ± 6.9
	n	-	0.0397 ± 0.0133
	R^2	-	0.9289
	AIC	-	84.55
Weibull	td	h	0.6 ± 0.2
	β	-	0.79 ± 0.44
	Q∞	μg	198.3 ± 3.8
	R^2	-	0,9425
	AIC	-	84.65
Higuchi	Kh	h^{-1}	33.9 ± 3.5
	R^2	-	-0.8166
	AIC	-	111.72

Equations S1–S5 describe the different kinetic models for drug release:

- Hyperbole model:

$$Q_t = \frac{Q_{max} \times t}{K_d + t} \quad \text{(S1)}$$

where, Qt = cumulative percentage of drug release at time t; Qmax = maximum amount of drug release; Kd = time required to reach 50% drug release.

- First-order model:

$$Q_t = Q_\infty \times (1 - e^{-kf \times t}) \quad \text{(S2)}$$

where, Q∞ = maximum amount of drug release; Kf = release rate constant.

- Korsmeyer-Peppas:

$$Q_t = K_k \times t^n \quad \text{(S3)}$$

where, Kk = release rate constant; n = release exponent.

- Weibull:

$$Q_t = Q_\infty \times \left[1 - e^{-(t/td)^{\beta}}\right] \quad \text{(S4)}$$

where, β = shape factor and td = time necessary to release the 63.2 % of the drug.

- Higuchi:

$$Q_t = K_H \times t^{1/2} \quad \text{(S5)}$$

where, K_H = release rate constant.
